# Genetic Predisposition for Type 2 Diabetes, but Not for Overweight/Obesity, Is Associated with a Restricted Adipogenesis

**DOI:** 10.1371/journal.pone.0018284

**Published:** 2011-04-12

**Authors:** Peter Arner, Erik Arner, Ann Hammarstedt, Ulf Smith

**Affiliations:** 1 Department of Medicine, Karolinska Institute at Karolinska Hospital, Huddinge, Stockholm, Sweden; 2 The Lundberg Laboratory for Diabetes Research, Center of Excellence for Metabolic and Cardiovascular Research, Department of Molecular and Clinical Medicine, the Sahlgrenska Academy at the University of Gothenburg, Gothenburg, Sweden; University of Tor Vergata, Italy

## Abstract

**Background:**

Development of Type 2 diabetes, like obesity, is promoted by a genetic predisposition. Although several genetic variants have been identified they only account for a small proportion of risk. We have asked if genetic risk is associated with abnormalities in storing excess lipids in the abdominal subcutaneous adipose tissue.

**Methodology/Principal Findings:**

We recruited 164 lean and 500 overweight/obese individuals with or without a genetic predisposition for Type 2 diabetes or obesity. Adipose cell size was measured in biopsies from the abdominal adipose tissue as well as insulin sensitivity (HOMA index), HDL-cholesterol and Apo AI and Apo B. 166 additional non-obese individuals with a genetic predisposition for Type 2 diabetes underwent a euglycemic hyperinsulinemic clamp to measure insulin sensitivity. Genetic predisposition for Type 2 diabetes, but not for overweight/obesity, was associated with inappropriate expansion of the adipose cells, reduced insulin sensitivity and a more proatherogenic lipid profile in non-obese individuals. However, obesity per se induced a similar expansion of adipose cells and dysmetabolic state irrespective of genetic predisposition.

**Conclusions/Significance:**

Genetic predisposition for Type 2 diabetes, but not obesity, is associated with an impaired ability to recruit new adipose cells to store excess lipids in the subcutaneous adipose tissue, thereby promoting ectopic lipid deposition. This becomes particularly evident in non-obese individuals since obesity per se promotes a dysmetabolic state irrespective of genetic predisposition. These results identify a novel susceptibility factor making individuals with a genetic predisposition for Type 2 diabetes particularly sensitive to the environment and caloric excess.

## Introduction

Type 2 diabetes is undergoing a global epidemic mainly driven by a concomitant epidemic in obesity [1]. The expanded adipose tissue mass in obesity promotes insulin resistance which, in turn, induces Type 2 diabetes in genetically susceptible individuals [2], [3]. It is also well-established that a preponderance of (intra)abdominal fat accumulation, estimated by an expanded waist or waist/hip circumference ratio, is a better marker of the obesity-associated complications, including the Metabolic Syndrome and cardiovascular disease than obesity per se [3], [4].

Accumulation of excess lipids in the adipose tissue can, in principle, be induced by an expansion of the number of differentiated adipose cells (hyperplastic obesity) and/or by adipose cell enlargement (hypertrophic obesity) [5], [6]. As a group, obese individuals have larger fat cells than non-obese [5], [6] and it has long been known that hypertrophic, rather than hyperplastic, obesity is closely associated with insulin resistance and the various aspects of the Metabolic Syndrome [5], [6] Interestingly, Spalding et al. [7] recently showed that the number of abdominal subcutaneous adipose cells becomes established around puberty and that adipose cell expansion becomes the predominant event for subsequent fat accumulation. However, adult women may still recruit new cells in the femoral/gluteal depot which appears to counteract abdominal adipocyte accumulation [8] and this may contribute to the well-established finding that peripheral (gluteo-femoral) obesity is less harmful than abdominal obesity [5], [6].

In order to differentiate between hypertrophic and hyperplastic obesity, Arner et al. [6] conceived the delta-factor as a quantitative statistical marker of inappropriate cell enlargement in relation to amount of body fat. A positive value indicates that fat cells are larger than expected from their body fat mass while a negative value indicates the opposite.

A major reason for the association between insulin resistance and abdominal adipose cell hypertrophy is probably the dysregulated adipose tissue with local insulin resistance, altered secretion of adipokines including reduced adiponectin levels and local infiltration of inflammatory cells which inhibits normal adipogenesis and differentiation of preadipocytes [9]–[12]. Other markers of the dysregulated adipose tissue include reduced levels of insulin signaling and action proteins like IRS-1 and GLUT4 protein in the abdominal adipose cells. This was initially described in patients with Type 2 diabetes [13], [14], but is now well established to precede the development of Type 2 diabetes and to be associated with insulin resistance and a dysmetabolic state also in non-obese individuals [10], [11], [15]. Interestingly, a dysregulated adipose tissue with inappropriate hypertrophy of the abdominal adipose cells was seen around four-times more commonly in individuals with a genetic predisposition for Type 2 diabetes [10], [11].

In the present study, we have measured adipose cell size, body fat and metabolic variables in a large group of individuals with or without a known genetic predisposition for overweight/obesity or Type 2 diabetes. The results clearly demonstrate that genetic predisposition for Type 2 diabetes, but not for overweight or obesity, is associated with inappropriate abdominal adipose cell enlargement in relation to amount of body fat. This becomes particularly evident in non-obese individuals since obesity per se promotes adipocyte expansion, and is associated with insulin resistance and its consequences. Thus, genetic predisposition for Type 2 diabetes is associated with adipose tissue dysfunction and insulin resistance long before obesity develops and indicates that this can be a major genetic susceptibility factor enhancing the negative effects of the environment and body fat increase.

## Materials and Methods

### Subjects

166 individuals with at least one known first-degree relative with Type 2 diabetes were recruited in Gothenburg and phenotyped as described [16]. In addition, 83 subjects with a genetic predisposition for Type 2 diabetes (first-degree relatives) were recruited in Stockholm. Leanness was defined as body mass index (BMI)<25 kg/m^2^. Seventeen of these individuals were lean and 66 overweight or obese (Table 1). In addition, 253 individuals with heredity for overweight/obesity (also first-degree relatives) were recruited; 56 of these were lean and 197 were overweight/obese when included. 91 lean and 237 overweight or obese individuals without any known genetic predisposition for these conditions were included for comparison (Table 1). Family history for overweight/obesity or Type 2 diabetes was determined as described [17].

**Table 1:** Comparison of lean and overweight individuals with or without a genetic predisposition for type 2 diabetes or overweight/obesity.

	Lean individuals				Overweight or obese individuals			
	Genetic predisposition				Genetic predisposition			
	Type 2 diabetes		Overweight or obesity		Type 2 diabetes		Overweight or obesity	
Measure	Yes(n = 17)	No(n = 65)	Yes(n = 56)	No(n = 26)	Yes(n = 66)	No(n = 184)	Yes(n = 197)	No(n = 53)
Age, years	38±2	33±1	35±1	32±1	40±1	40±1	40±1	38±1
Waist circumference, cm	82±2(*)	79±1	80±1*	77±1	117±2	114±1	116±1***	107±2
BMI, kg/m^2^	22.9±0.4	22.4±0.2	22.7±0.2	22.2±0.3	40.0±0.8*	37.0±0.5	38.3±0.4***	34.4±0.9
Body fat mass, kg	19±1	18±1	18±1	17±1	61±2	56±1	60±1***	48±3
Fat cell volume, pL	511±45**	400±19	431±23	407±30	824±21	806±14	827±12**	739±29
Delta value, pL	64±38**	−37±18	−15±21	−18±28	9±17	22±11	21±11	13±21
HOMA, index	1.62±0.24*	1.17±0.08	1.26±0.09	1.26±0.16	3.7±0.2	3.7±0.2	3.8±0.2	3.2±0.4
P-HDL cholesterol, mM	1.39±0.10*	1.62±0.05	1.56±0.07	1.60±0.07	1.17±0.05	1.20±0.02	1.19±0.02	1.26±0.05
P-Apo A1, mM	1.37±0.07	1.48±0.05	1.43±0.05	1.51±0.07	1.29±0.04	1.27±0.02	1.28±0.02	1.26±0.03
P-Apo B, mM	0.94±0.06(*)	0.82±0.04	0.86±0.04	0.84±0.06	1.03±0.04	1.03±0.03	1.03±0.02	1.05±0.06
P-ApoB/A1	0.72±0.07*	0.57±0.03	0.63±0.04	0.56±0.04	0.84±0.05	0.82±0.02	0.83±0.02	0.83±0.04

Values are mean ± SE. Significances (by t-test) were only calculated between groups with heredity or not for type 2 diabetes and between groups with heredity or not for overweight or obesity.

(*)0.05<p<0.1,

*p<0.05,

**p = 0.01,

***p<0.001.

### Ethics statements

The studies were approved by the local Ethical Committees at the Sahlgrenska Academy and the Karolinska Institute and were performed in agreement with the Declaration of Helsinki. All subjects received written information and gave written consents to participate.

### Methods

Adipose tissue biopsies were obtained from the abdominal subcutaneous adipose tissue around the umbilicus. Following careful dissection, the adipose cells were digested with collagenase and cell size measured with an ocular as previously reported [6], [12]. The methods used in the laboratories of Stockholm and Gothenburg were carefully compared and validated as similar. However, to exclude any bias, the effect of the genetic background on cell size was only evaluated in the Stockholm cohorts (Table 1). The delta-values were calculated [6] as these values of adipose morphology are independent of body fat mass. The values are quantitative, i.e., a large negative value indicates pronounced hyperplasia and a large positive value indicates pronounced hypertrophy [6]. The unadjusted mean average fat cell volume in each individual was also investigated and related to the amount of body fat. All subjects underwent bioimpedance measurements of body composition and waist circumference was measured [5], [6]. Blood samples were drawn for measurements of fasting lipids, glucose and insulin levels as reported [6], [16] and HOMA index, which is an indirect measure of insulin sensitivity, was calculated [18]. Euglycemic hyperinsulinemic glucose clamps were performed in the Gothenburg co-hort comprising 166 individuals with known heredity for Type 2 diabetes as previously reported [16] and the results were related to average abdominal cell volume or the delta-values.

### Statistical analysis

Significances of differences were calculated with unpaired t-test and conventional methods were used to calculate means ± standard error of mean (SE). Analysis of co-variance (ANCOVA) was used to analyse statistical significance of cell volume differences of subjects with or without a heredity for Type 2 diabetes or overweight/obesity.

## Results

### Fat cell size in relation to body fat

**Figure 1:**
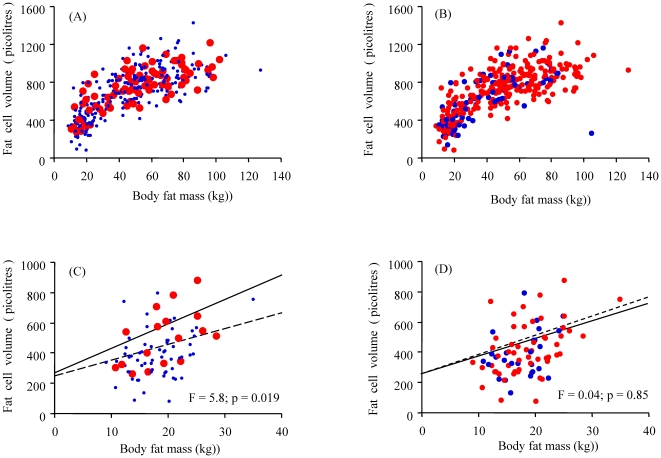
Body fat in relation to adipose cell size. Adipose cell size in relation to body fat in all individuals with (red circles) or without (blue dots) a known genetic predisposition for Type 2 diabetes (A) or overweight/obesity (B). The figures below display the relationships between fat cell size and body fat in lean individuals (BMI<25 kg/m^2^ with (solid lines) or without (broken lines) a diabetes (C) or overweight/obesity heredity (D). Significance of differences between the groups in cell size vs body fat was tested with ANCOVA for those with or without diabetes (F = 5.8, p = 0.019) (C) or overweight/obesity (F = 0.04, p = 0.85) (D) heredity.

As shown in Figure 1A for all investigated subjects with/without a known genetic predisposition for Type 2 diabetes, several lean individuals with a known genetic predisposition for Type 2 diabetes showed a marked increase in abdominal fat cell volume over a small increase in body fat as compared with lean subjects without diabetes heredity. When lean subjects (BMI<25 kg/m^2^) were analyzed separately this influence of diabetes heredity was significant by ANCOVA (Figure 1C) (F = 5.8; p = 0.019). In contrast, in the whole sample as well as in a separate analysis, lean individuals with heredity for overweight/obesity had the same relationship between fat cell volume and fat mass as lean subjects without such heredity (Figure 1B and 1D) (F = 0.04 NS). Thus, a known genetic predisposition for Type 2 diabetes, but not for a high BMI, leads to inappropriately enlarged fat cells even when the subjects are considered lean. In fact, many lean individuals with a diabetes heredity can from the point of view of their fat cell size be considered as obese.

### Phenotypic characterization

**Figure 2:**
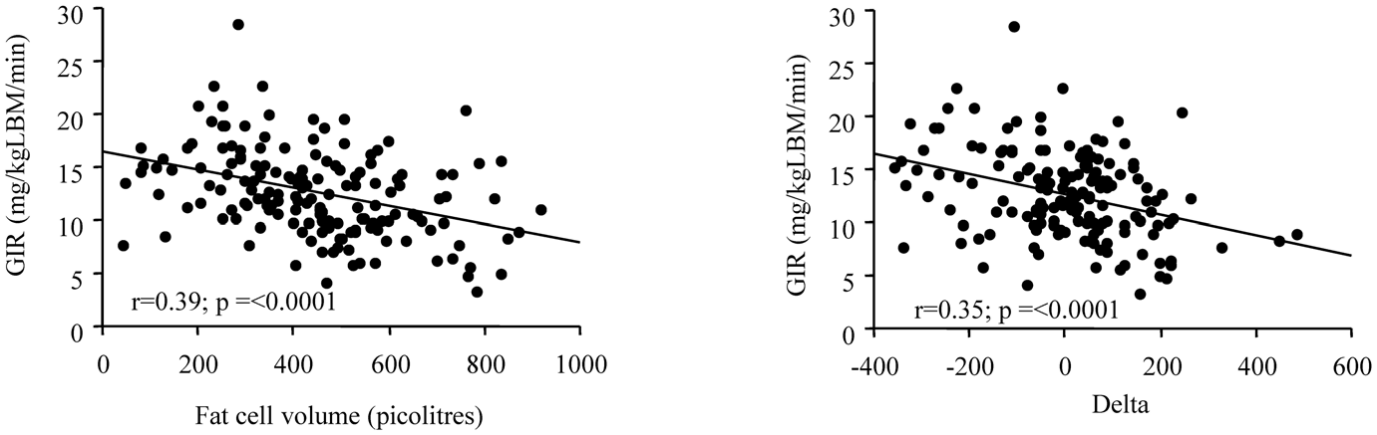
Insulin sensitivity in relation to adipose cell size. (A) Insulin sensitivity, measured with the euglycemic clamp, in non-obese individuals (BMI<28 kg/m^2^) with a genetic predisposition for Type 2 diabetes in relation mean abdominal adipose cell volume (r = −0.39, p<0.0001) and (B) delta-values (r = 0.35, p<0.0001). The results are expressed as mg glucose infused (GIR)/kg lean body mass (LBM) and minute.

We also examined abdominal adipose cell size in relation to insulin sensitivity measured with a euglycemic clamp in the Gothenburg group of 166 non-obese individuals (BMI<28 kg/m^2^) with a known genetic predisposition for Type 2 diabetes. As shown in Figure 2, fat cell sizes which are considered within the normal range in individuals lacking a diabetes heredity (<800 pl, which was upper limit for lean with no diabetes heredity in Figure 1A) were associated with a gradual reduction in insulin sensitivity. This was also true when the relationship between insulin sensitivity and inappropriately enlarged fat cells (for a given BMI) was expressed as the delta-values (Figure 2B). These results are also consistent with the concept that lean individuals with diabetes heredity exhibit an “obese” phenotype with inappropriately enlarged fat cells associated with a reduced insulin sensitivity.

We further verified the results by comparing HOMA-derived insulin sensitivity and different metabolic variables in lean and obese individuals with or without a genetic predisposition for Type 2 diabetes or overweight/obesity (Table 1). Individuals with a genetic predisposition to Type 2 diabetes again showed a reduced insulin sensitivity as well as a more pro-atherogenic profile when compared to the individuals lacking heredity for diabetes (larger waist circumference, lower HDL-cholesterol, higher Apo B and Apo B/Apo A1 ratios) (Table 1). Consistent with the results in Figure 1A, the delta-value as a marker of inappropriate cell enlargement, was also significantly increased in non-obese individuals with heredity for Type 2 diabetes (p = 0.01). Furthermore, the average unadjusted fat cell volume was increased in those with a diabetes heredity in spite of no difference in fat mass or BMI compared to these with a genetic predisposition for overweight/obesity.

We also extended the analysis to include individuals with a BMI up to 27 kg/m^2^. However, essentially the same results were found since heredity for Type 2 diabetes was again associated with larger adipose cells when compared to matched subjects lacking a known genetic predisposition or having heredity for obesity (adipose cell size 506±42 pL vs 410 pL, p<0.05). Similarly, the delta-value was significantly higher in the subjects with a diabetes heredity (p<0.01). These results further corroborate the concept that genetic predisposition for Type 2 diabetes is associated with an impaired ability to recruit preadipocytes to store excess fat in the subcutaneous adipose tissue. This becomes most prominent in non-obese individuals since obesity per se promotes adipose cell expansion towards an upper maximal level.

We further validated that this difference was specifically related to a genetic predisposition for Type 2 diabetes and not for overweight or obesity. Individuals with a genetic predisposition for a high BMI showed no evidence of an inappropriate expansion of cell size in relation to amount of body fat measured as delta-value (Table 1). In addition, heredity for a high BMI was not associated with an adverse insulin-metabolic phenotype in lean individuals in contrast to those with a diabetes heredity. Taken together, these results show that a genetic predisposition for Type 2 diabetes, but not for a high BMI, is associated with an “obese” phenotype in lean individuals and they also exhibit a more pro-atherogenic metabolic profile. However, obesity per se promotes both adipose cell expansion and a dysmetabolic state to a similar extent irrespective of heredity for Type 2 diabetes or overweight/obesity.

## Discussion

These results show that a genetic predisposition for Type 2 diabetes in non-obese individuals is associated with inappropriate expansion of the abdominal adipose cells for small increases in body fat. Thus, they assume an “obese” phenotype long before the conventional definition of overweight or obesity. In contrast, genetic predisposition for a high BMI, although promoting an obese phenotype, is not associated with inappropriate abdominal adipose cell enlargement in relation to amount of body fat when measured as absolute size or delta-values. Insulin sensitivity is also reduced and negatively correlated to adipose cell size in non-obese subjects with heredity for Type 2 diabetes. Taken together, these data suggest that a genetic predisposition for Type 2 diabetes is associated with an impaired ability to recruit and/or differentiate new adipose cells to store excessive lipids. This finding is consistent with our previous results that diabetes heredity in non-obese subjects is frequently associated with markers of a dysregulated adipose tissue, inappropriate adipose cell hypertrophy and reduced circulating adiponectin levels [10], [11]. Also other markers of a dysregulated adipose tissue, including markers of terminal cell differentiation and PPARγ-regulated genes such as adiponectin and GLUT4, are commonly reduced in the abdominal adipose tissue in individuals with a genetic predisposition for Type 2 diabetes [10], [11], [15]. The concept of an impaired recruitment of new and functional adipose cells in individuals with a genetic predisposition for Type 2 diabetes, leading to inappropriately enlarged abdominal adipose cells, is further supported by recent prospective studies showing that enlarged fat cells predispose for future development of Type 2 diabetes irrespective of BMI [19], [20].

The molecular mechanisms for the impaired recruitment and/or commitment of new subcutaneous preadipocytes in individuals with a genetic predisposition for Type 2 diabetes are currently unclear. Likely possibilities include maintained canonical Wnt activation and/or the infiltration of macrophages and inflammation in the adipose tissue. TNFα would appear as an interesting candidate since it is a powerful inhibitor of differentiation of human preadipocytes [12] and, in addition, activates Wnt signaling [21]. In this regard, potential genetic factors would also include altered activity of molecules cleaving and activating TNFα like the TNF converting-enzyme [22].

Taken together our findings provide a novel mechanism whereby a genetic predisposition for Type 2 diabetes makes the individuals particularly susceptible to the environment and that even small increases in body fat promote a reduced insulin sensitivity and a dysmetabolic and proatherogenic phenotype with an increased risk to develop Type 2 diabetes. Thus, diabetes heredity is associated with an increased susceptibility to the environment in terms of becoming “obese” in the adipose tissue even when they have small amounts of body fat. If this inappropriate expansion of adipose cell size is related to a reduced stem cell commitment and/or subsequent differentiation of committed preadipocytes is unknown but a current focus of study.
